# The abnormalities of brain function in females with primary insomnia: a resting-state functional magnetic resonance imaging study

**DOI:** 10.3389/fnins.2024.1414154

**Published:** 2024-07-31

**Authors:** Haiyi Zhang, Pingping Jie, Yingchun Liu, Lunxin Wu, Oucheng Wang, Yong Zhang, Jiliang Fang, Quan Wang, Jie Zhao, Yong Liu

**Affiliations:** ^1^Department of Magnetic Resonance Imaging, The Affiliated Traditional Chinese Medicine Hospital, Southwest Medical University, Luzhou, Sichuan, China; ^2^Department of Acupuncture, Moxibustion, Tui-Na and Rehabilitation, The Affiliated Traditional Chinese Medicine Hospital, Southwest Medical University, Luzhou, Sichuan, China; ^3^Guang’anmen Hospital, China Academy of Chinese Medical Sciences, Beijing, China; ^4^Department of General Family Medicine, The Affiliated Traditional Chinese Medicine Hospital, Southwest Medical University, Luzhou, Sichuan, China

**Keywords:** primary insomnia, females, resting-state functional magnetic resonance imaging, functional connectivity, neuropathologic mechanism

## Abstract

**Background:**

The neuropathologic mechanism of primary insomnia (PI) of females remains unclear. This study aims to investigate the features of amplitude of low-frequency fluctuations (ALFF) and regional homogeneity (ReHo) in females with PI using functional magnetic resonance imaging (fMRI), and then explore the abnormalities of functional connectivity (FC).

**Materials and methods:**

A total of 39 female PI patients and 31 female healthy controls (HC) were enrolled in the study. The sleep condition was assessed using the Pittsburgh Sleep Quality Index (PSQI), and Insomnia Severity Index (ISI), and their depressive symptom was evaluated using the Hamilton Depression Scale (HAMD-24). The rs-fMRI was once conducted for every subject. ReHo, ALFF, and ROI-based FC were used to analyze the changes of brain function.

**Results:**

ALFF values were increased in the Cerebelum_4_5_L, as well as decreased ALFF in the bilateral Frontal_Sup_Medial (SFGmed), Angular_L (ANG.L), Parietal_Inf_R (IPL.R), SupraMarginal_R (SMG.R), and Postcentral_R (PoCG.R). ReHo values were increased in the Temporal_Pole_Mid_R (TPOsup.R), as well as decreased ReHo in the Insula_R (INS.R), Frontal_Inf_Oper_R (ORBinf.R), Putamen_R (PUT.R), Rolandic_Oper_R (ROL.R), bilateral Cingulum_Post (PCG), bilateral Frontal_Sup_Medial (SFGmed), bilateral anterior cingulate and paracingulate gyri (ACG), and the bilateral precuneus (PCUN). Across the entire brain, there was a decline in the FC between Angular_R and Frontal_Sup_Medial_L.

**Conclusion:**

Alterations in brain regions of female patients with PI involved multiple functional networks, including the default mode network, the salience network, the central executive network, and the limbic network. Reduced coordination between functional networks may be an important mechanism for insomnia and may lead to reduced cognitive function and decision-making ability.

## 1 Introduction

A mental condition known as primary insomnia (PI) is characterized by difficulties falling asleep, staying asleep, waking up frequently, and waking up early enough to interfere with re-entry and produce dysfunction and misery during the day (Morin et al., 2015). Previous survey indicated that the global prevalence of insomnia symptoms is estimated to be approximately 30–35%, yet only a small proportion of cases were successfully identified and managed (Morin et al., 2015). A persistently negative mindset can be fostered by persistent insomnia since it can gradually cause a lack of confidence and control over sleep (Blanken et al., 2020; Goldstone et al., 2020). Persistent insomnia may also cause patients’ cognitive function to deteriorate, raising their risk of depression and suicide (Chattu et al., 2019). Prolonged sleep insufficiency may also result in various systemic diseases, exerting a considerable adverse influence on patients’ quality of life and causing varying degrees of impairment to their physical and mental well-being (Morin et al., 2015). In brief, the main scientific problems facing the clinic today are unknown mechanisms and subjective diagnoses.

Clinical studies on PI imply that abnormal emotional or psychological alterations exist in these patients (Baglioni et al., 2010). Electrophysiologic studies of insomnia have shown smaller declines in glucose metabolism from sleep condition to wakefulness in mood-related brain regions (Nofzinger et al., 2004). Cano et al. (2008) found similar features in the rat model as well. For imaging studies, structural magnetic resonance imaging (sMRI) and diffusion tensor imaging (DTI) technology were used to explore the alterations in gray matter volume and white matter microstructure in abnormal brain regions of PI patients, which indicated that some brain regions with emotion control function were changed (Stoffers et al., 2012; Wu et al., 2018). These studies furnish certain evidence indicating that insomnia leads to neurological alterations. However, the neurobiological mechanisms underlying PI are not fully clear. The neural mechanisms underlying the intrinsic link between insomnia and mood changes have not been elucidated.

Functional magnetic resonance imaging (fMRI) can indirectly reflect the function of the brain and is a useful tool for studying the neurobiological causes and clinical manifestations of PI (Sun et al., 2022). Blood oxygen level-dependent (BOLD) signals have been the basis for the widespread use of resting-state fMRI (rs-fMRI) in recent years for the study of psychiatric disorders, including attention-deficit hyperactivity disorder, schizophrenia, bipolar disorder, and autism spectrum disorders (Gao et al., 2020; Sun et al., 2022; Zhao et al., 2022). Using measurements of the magnetic fields of deoxyhemoglobin and the magnetic characteristics of oxyhemoglobin to track the level of neuronal activity in particular brain regions, rs-fMRI is mostly used in investigating the impact of insomnia on the brain’s functioning (Ji et al., 2021; Raimondo et al., 2021; Zhang et al., 2021). In the investigation of changes in brain function in individuals with PI, rs-fMRI offers a more comprehensive understanding (Yang et al., 2023). Previous research has demonstrated the potential utility of rs-fMRI in the investigation of PI. For instance, in patients with insomnia, previous studies have disclosed elevated ReHo values in the right superior frontal gyrus and decreased ReHo values in the left cerebellar gyrus, left suboccipital gyrus, and left amygdala (Zhang et al., 2021). It has also indicated gender disparities in the prevalence of PI, with consistently higher rates observed in females across different age groups (Suh et al., 2018). A systematic review suggests that variations in neurohormone secretion, biological processes, and brain morphology between genders may contribute to these differences, warranting further investigation into underlying mechanisms (Pajėdienė et al., 2024). Therefore, a comprehensive understanding of sex-specific symptomatology and pathological pathways is crucial for effective prevention and intervention strategies.

However, limited attention has been given to investigating the neuroimaging findings associated with characteristics in female individuals with PI. Previous studies have primarily focused on gender differences without delving into the specific traits of female patients (Kocevska et al., 2021; Pajėdienė et al., 2024). We guess that the high prevalence of PI cases in females may be attributed to unique activity of brain region. In this study, we conducted a comparative analysis of brain region activity in female PI patients and healthy females to investigate potential characteristic and further reveal the underlying regularity of neuronal activity.

## 2 Materials and methods

### 2.1 Participants

A total of 39 patients with PI as well as 31 healthy controls (HC) were included in the study. All participants were collected from the outpatient clinic of Affiliated Traditional Chinese Medicine Hospital, Southwest Medical University from 2022 to 2023. None of the participants were menstruating at the time of the scan, and all had never taken sleep drugs or stimulants in the days before the fMRI session. All participants confirmed that they did not fall asleep during the scan. Patients were included in this study if they met the following criteria: (1) met the diagnostic criteria for PI in the U.S. Diagnostic and Statistical Manual of Mental Disorders, Fifth Edition (DSM-V) (Wakefield, 2016); (2) had various forms of insomnia as the main symptom, with other symptoms secondary to insomnia; (3) female, 25–70 years of age; (4) right-handed; (5) Pittsburgh Sleep Quality Index (PSQI) score ≥ 8; Hamilton Depression Scale (HAMD-24) score ≤ 8, Insomnia Severity Index (ISI) score ≥ 8. excluded if: (1) abnormal signals confirmed by routine T1 or T2 fluid-attenuation inversion recovery (FLAIR) MRI; (2) insomnia caused by direct physical or mental or general illness; (3) pregnancy; (4) taking sedative-hypnotic medication or stimulants such as staying up all night, drinking alcohol, tea, and coffee; and (5) contraindications to MRI.

Participants in the HC group were relatively matched in terms of gender, age, and education level, and the inclusion criteria were: (1) female, 25–70 years of age; (2) right-handed; (3) good quality of sleep with no history of staying up all night for 1 week; (4) no psychiatric or neurological disorders; (5) no MRI contraindications and no abnormal signals confirmed by routine T1 or T2 FLAIR; (6) PSQI <8 points, HAMD-24 ≤ 8 points, ISI < 8 points; and (7) no history of staying up all night, drinking large amounts of alcohol, drinking tea, coffee, and other stimulating foods within 2 days before MRI examination.

All subjects gave written informed consent in accordance with the Declaration of Helsinki. The study was approved by the Ethics Committee of the Affiliated Hospital of Traditional Chinese Medicine of Southwest Medical University (KY2021082-FS01).

### 2.2 Measurements

Based on prior research findings, the PSQI, ISI, and HAMD-24 scales were employed as instruments for evaluating the sleep quality and emotional state of patients (Bastien et al., 2001; Zhang et al., 2021; Lin et al., 2022). All participants accepted PSQI and ISI before the 30 min of the scan. To exclude the risk of depression, which may independently affect imaging findings, we used HAMD to estimate the mental status of all the participants.

### 2.3 MRI scan

Siemens Skyra 3.0 T MRI (Siemens Magnetom Verio; Siemens Medical Systems, Erlangen, Germany) with a 16-channel combined head and neck coil was used for data acquisition, and routine T1W, T2W, T2-FLAIR, and other scans were performed before resting-state data acquisition to exclude patients with cerebral hemorrhage and tumors. T1WI, T2WI, T2-FLAIR, and other sequential scans were performed before resting-state data acquisition to exclude cerebral hemorrhage, infarction, and tumor. During the resting-state scanning, the subjects were asked to lie down quietly, breathe calmly with eyes closed, try not to perform any thinking movement, fix the head with foam pads to reduce head movement and wear earplugs to reduce the noise, and the scanning was started after the subjects were familiarized with the environment. Functional imaging of resting cerebral oxygen-dependent levels was acquired using a gradient recalled echo (GRE) sequence with the following parameters: transition time (TR) 2,720 ms, echo time (TE) = 40 ms, flip angle 90°, thickness/gap = 4.0/0 mm, field of view (FOV) = 240 mm × 240 mm, in-plane resolution = 64 × 64, 38 axial slices, acquisition time point 270, and acquisition times of 12 min and 20 s. In addition, anatomical T1-weighted whole brain magnetization-prepared rapid gradient-echo (MPRAGE) images were acquired with the following parameters: repetition time (TR) 1960 ms, echo time (TE) = 2.98 ms, flip angle 90°, thickness/gap = 1.0/0 mm, field of view (FOV) = 256 mm × 256 mm, in-plane resolution = 256 × 256, and number of sagittal slice layers 176.

### 2.4 Image analysis

Preprocessing was based on the MATLAB R2013b platform using RESTplus1.2 software (REST; http://restfmri.net). (1) Removal of data from the first 10 time points, subsequent 260 time points of fMRI data were included in the analysis. The signal fluctuates at the beginning of the scan, the process of adapting to the scanning environment and thus the magnetic field is unstable, and the removal of the data from the first 5 time points is needed to reduce or eliminate the influence of these data on the results; (2) slice timing; (3) realign: exclude images of subjects with head movement translations >2.5 mm and rotational shifts >2.5°; (4) normalization: align BOLD images with the corresponding T1 structural images, followed by spatial normalization using the standard Montreal Neurological Institute and Hospital (MNI) spatial template for spatial normalization; (5) Resampling of the functional image voxel size to 3 × 3 × 3 mm; (6) spatial smoothing [ReHo was analyzed first and then smoothed, ALFF was smoothed first, and the smoothing kernel (FWHM) for spatial smoothing was set to 6 × 6 × 6 mm]; (7) detrend; (8) nuisance covariates regression: the effect of white matter mean signal, cerebrospinal fluid mean signal, and head movement on the data; and (9) to eliminate high-frequency noise, a band-pass filter was applied to the data, keeping the frequency between 0.01 and 0.08 Hz.

Subsequently, we refer to the method proposed by Wang et al. (2022) and Zang et al. (2004). The RESTplus software’s Postprocessing module was used to examine the data for ReHo, ALFF, and FC. The step of analysis Kendall consistency coefficients (KCC) between voxels around their closest 26 neighboring voxels were determined using ReHo analysis and divided by the average KCC of the entire brain to reduce the impact of individual variations on KCC values. Lastly, a 6 mm FWHM was used to Gaussian smooth the data to reduce the impact of noise and anatomical variations. Using the fast Fourier transform, the filtered time series of each voxel in the subject’s brain was transformed into a frequency spectrum, and the power spectrum was produced. The ALFF was then calculated by taking the square root of the power spectrum between 0.01 and 0.08 Hz, and each voxel’s ALFF was divided by the global mean of the ALFF to obtain the standardized zALFF for further statistical analysis. Regions of interest (ROIs) were then created based on the brain region where the peak point is located. Use these brain regions where changes exist as seed points (seeds). The average time series of all the voxels in each ROI was then extracted, and the resting-state fMRI data analysis toolkit (RESTplus) was used to compute Pearson’s correlation coefficients between each ROI (seed to seed). Brain regions were delineated using the AAL template. Finally, Fisher’s Z-transform was used to smooth the data and return it to a more normal distribution.

### 2.5 Statistical analysis

The statistical program IBM SPSS 26.0 (IBM, Chicago, IL, United States) was used to examine the subjects’ basic data from both groups. For ages, which followed a normal distribution, a *t*-test for independent samples was applied. The Mann–Whitney *U* test was utilized to examine the remaining measures, which included years of education, PSQI, ISI, and HAMD-24 scale scores. Using the preprocessed fMRI data mentioned above, two-sample *t*-tests were conducted using SPM12.0 software (Statistical Parametric Mapping 12, http://www.fil.ion.ucl.ac.uk/spm/software/spm12/). The FWE method was chosen to account for multiple comparisons, and regions with a corrected *p* < 0.05 were deemed statistically significant regions. The MNI coordinate system (Montreal) for peak points in the statistically significant regions of difference was employed. The MNI coordinates of the peak points in the regions with statistically significant differences were localized, and the range of brain regions with larger voxel levels was selected and named as statistically significant brain regions. The count of FC values was analyzed through univariate linear regression using IBM SPSS 26.0 statistical software.

## 3 Results

### 3.1 General clinical characteristics

This study included 39 female PI patients and 31 female HC participants. The results showed that there was no statistically significant difference between the female PI group and the HC group in terms of age and education level (*p* > 0.05). However, the PSQI, HAMD scale, and ISI scale scores of the PI group were significantly higher than those of the control group, and the difference was statistically significant (*p* < 0.001) (Table 1).

**Table 1:** General clinical characteristics.

	PI group (PI)	Healthy control (HC)	*T/Z* value	*p* value
Age, year^a^	48.38 ± 1.63	44.68 ± 1.69	1.562^c^	0.123
Edu, year^b^	9.41 ± 3.69	11.16 ± 5.51	−1.507^d^	0.132
PSQI^b^	14.20 ± 1.63	3.42 ± 1.20	−7.050^d^	<0.001
ISI^b^	20.90 ± 3.56	2.13 ± 1.23	−7.174^d^	<0.001
HAMD-24^b^	6.33 ± 0.90	1.25 ± 0.62	−7.360^d^	<0.001

Unless otherwise indicated, data are mean ± standard deviation.

a Student’s *t*-test.

b Mann–Whitney U test.

c *T* value.

d Z value.

ISI: Insomnia Severity Index. PSQI: Pittsburgh Sleep Quality Index; HAMD, Hamilton Depression Scale.

### 3.2 ALFF changes in PI patients

**Figure 1:**
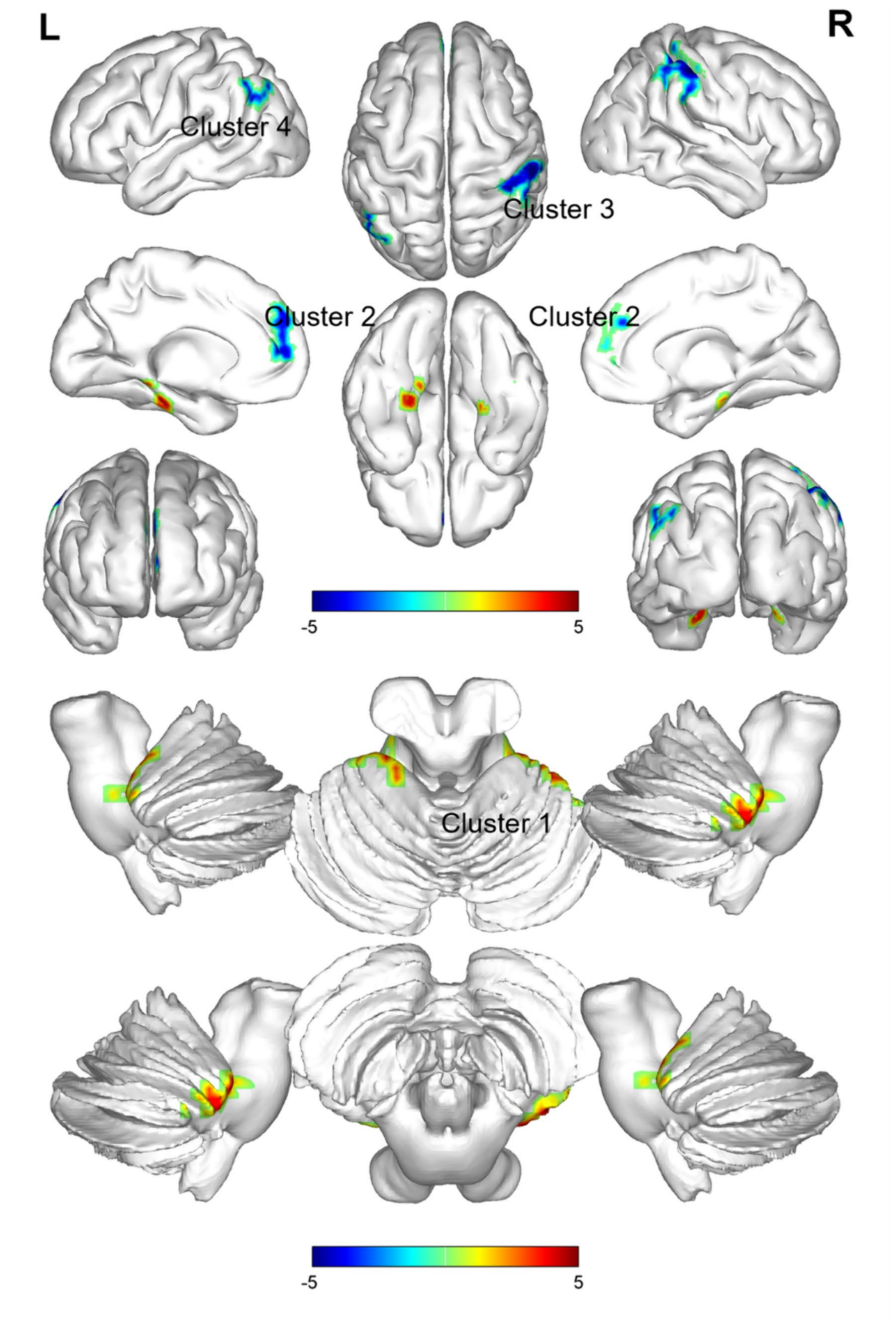
Brain regions (Cerebelum_4_5_L, Frontal_Sup_Medial_L, Frontal_Sup_Medial_R, Parietal_Inf_R, SupraMarginal_R, Postcentral_R, Angular_L) showing significant ALFF in the PI group compared with the HC group on the 3D template (*p* < 0.05, GRF corrected). The color scale represents the *t*-value. Cool colors (blue) indicate a significant decrease in value; warm colors (red) indicate a significant increase in value. R = right, L = left.

Comparison between the PI group and the HC group showed that 7 brain regions differed between the two groups (*p* < 0.05, GRF corrected, Table 2). ALFF values were increased in Cerebelum_4_5_L (Figure 1), and decreased in Frontal_Sup_Medial_L, Frontal_Sup_Medial_R, Parietal_Inf_R, SupraMarginal_R, Postcentral_R, Angular_L (Figure 1).

**Table 2:** Differential brain regions based on ALFF between PI and HC.

Brain regions	Voxel^a^ (mm^3^)	AAL	MNI coordinates			*T* value
			x	y	z	
**PI>HC**						
**Cluster 1**	49		−24	−27	−30	4.69
Cerebelum_4_5_L	22	97				
**PI<HC**						
**Cluster 2**	111		−3	54	9	4.45
Frontal_Sup_Medial_L	57	23				
Frontal_Sup_Medial_R	20	24				
**Cluster 3**	220		57	−30	48	5.51
Parietal_Inf_R	89	62				
SupraMarginal_R	72	64				
Postcentral_R	35	58				
**Cluster 4**	50		−48	−57	39	3.86
Angular_L	37	65				

a We chose clusters with voxel levels greater than 20 mm^3^.

### 3.3 Reho changes in PI patients

**Figure 2:**
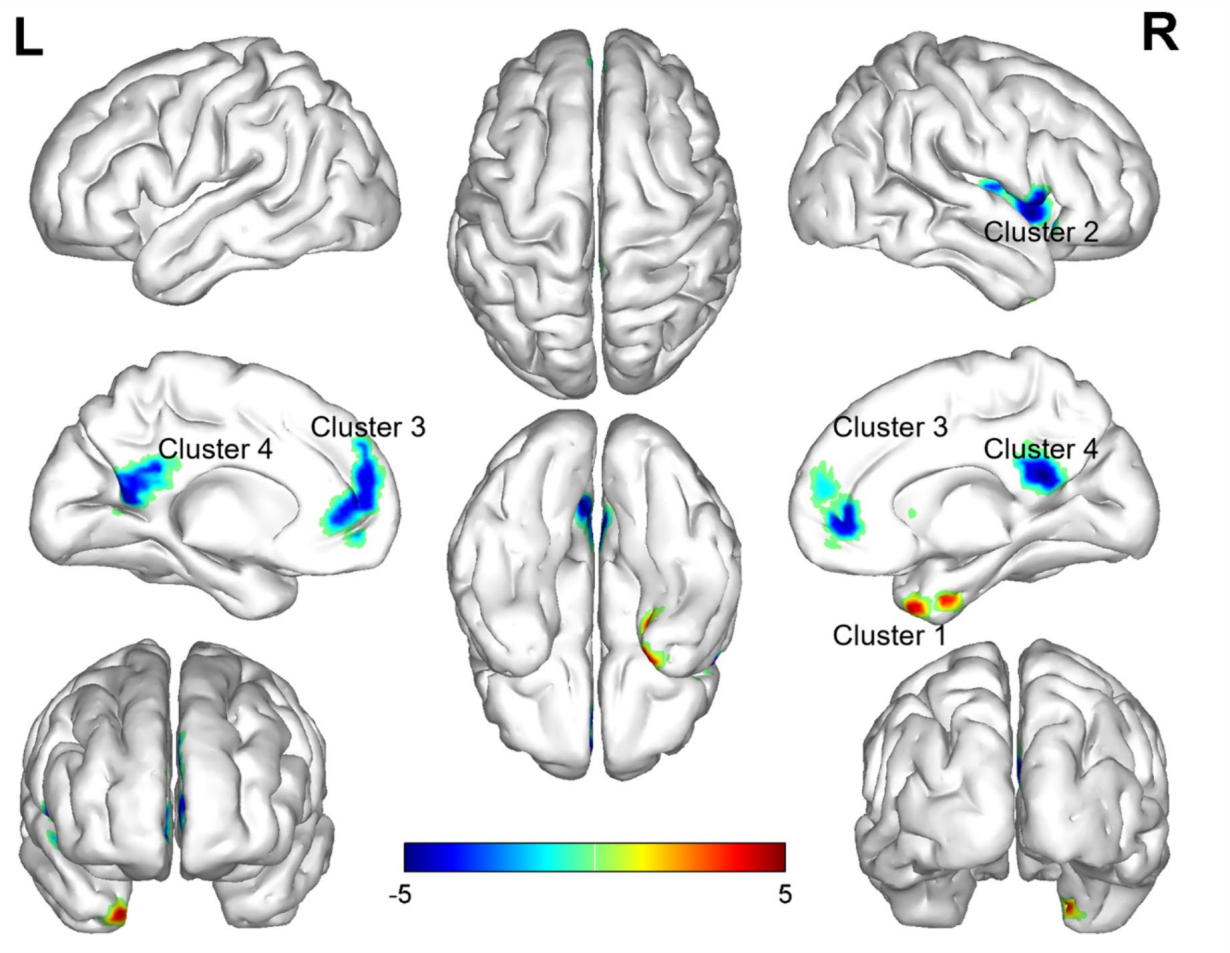
Brain regions (Temporal_Pole_Mid_R, Insula_R, Frontal_Inf_Oper_R, Putamen_R, Rolandic_Oper_R, Frontal_Sup_Medial_L, Cingulum_Ant_L, Frontal_Sup_Medial_R, Cingulum_Ant_R, Cingulum_Post_L, Precuneus_L, Cingulum_Post_R, Precuneus_R) showing significant ReHo in the PI group compared with the HC group on the 3D template (p < 0.05, GRF corrected). The color scale represents the *t*-value. The color scale represents the *t*-value. Cool colors (blue) indicate a significant decrease in value; warm colors (red) indicate a significant increase in value. R = right, L = left.

In the comparison of ReHo values between the two groups, significant differences were observed in a total of 13 brain regions (*p* < 0.05, GRF corrected, Table 3). ReHo values were increased Temporal_Pole_Mid_R (Figure 2), and decreased in Insula_R, Frontal_Inf_Oper_R, Putamen_R, Rolandic_Oper_R, Frontal_Sup_Medial_L, Cingulum_Ant_L, Frontal_Sup_Medial_R, Cingulum_Ant_R, Cingulum_Post_L, Precuneus_L, Cingulum_Post_R, Precuneus_R (Figure 2).

**Table 3:** Differential brain regions based on ReHo between PI and HC.

Brain regions	Voxel^a^ (mm^3^)	AAL	MNI coordinates			*T* value
			x	y	z	
**PI>HC**						
**Cluster 1**	69		30	0	−36	4.51
Temporal_Pole_Mid_R	40	84				
**PI<HC**						
**Cluster 2**	223		48	15	−6	5.58
Insula_R	76	30				
Frontal_Inf_Oper_R	48	12				
Putamen_R	30	74				
Rolandic_Oper_R	28	18				
**Cluster 3**	190		6	45	−3	4.43
Frontal_Sup_Medial_L	68	23				
Cingulum_Ant_L	47	31				
Frontal_Sup_Medial_R	27	24				
Cingulum_Ant_R	21	32				
**Cluster 4**	202		6	−45	21	5.58
Cingulum_Post_L	65	35				
Precuneus_L	55	67				
Cingulum_Post_R	42	36				
Precuneus_R	21	68				

a We chose clusters with voxel levels greater than 20 mm^3^.

### 3.4 Altered FC of ROI-wise brain regions

**Figure 3:**
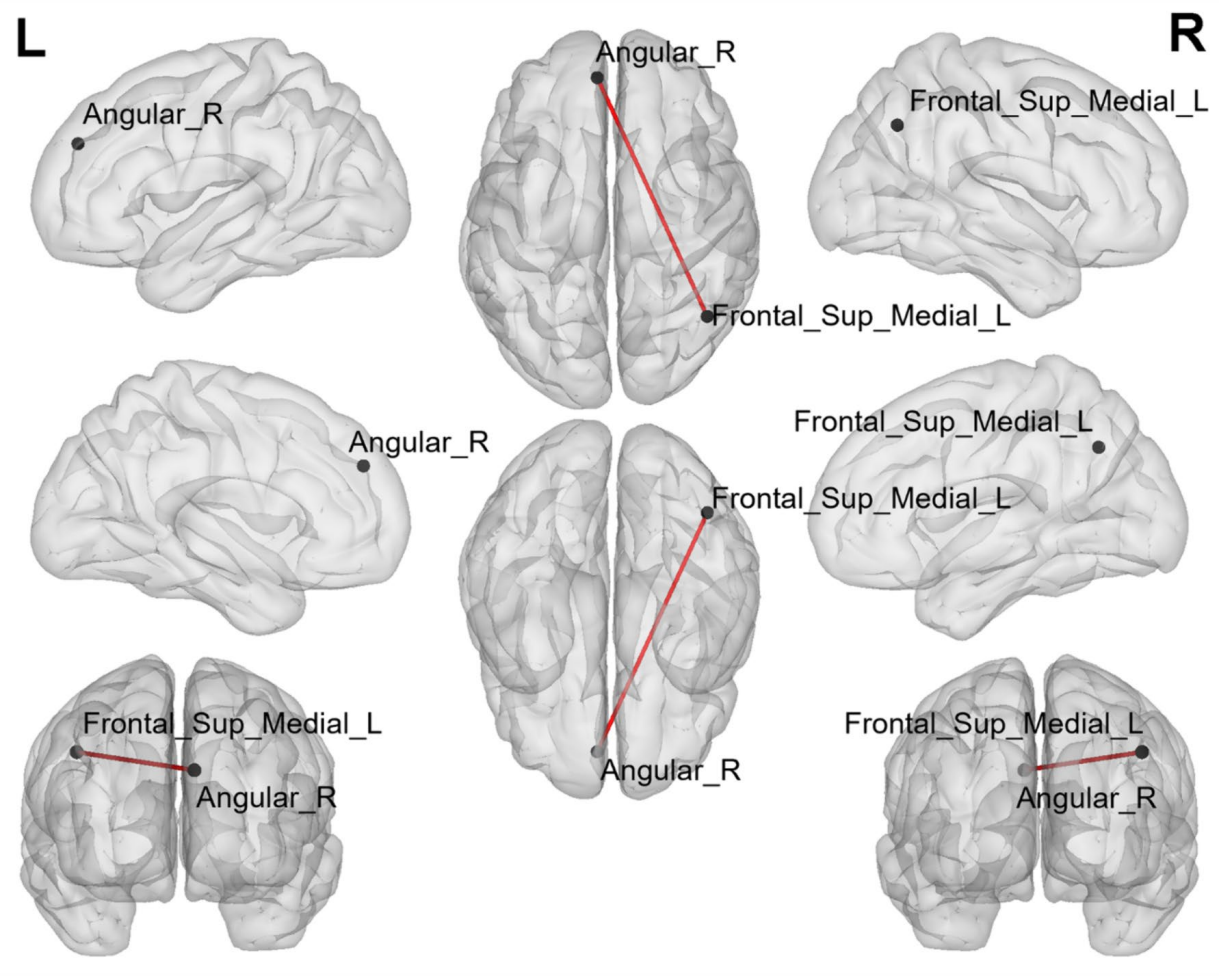
The black dots represent brain ROI, and the red lines represent brain regions that show a decline in functional brain connectivity (*p* < 0.05/28, bonferroni corrected). R = right, L = left.

In ROI-wise FC, a total of 1 group of brain region connectivity were abnormal in the whole brain (*p* < 0.05/28, bonferroni corrected, Figure 3; Table 4), and this group of brain region connectivity in the whole brain decreased.

**Table 4:** Altered functional connectivity of ROI-wise brain regions (seed-seed).

ROIs (seeds)	Brain regions (seeds)	*F* value	*p* value
Angular_R	Frontal_Sup_Medial_L	11.473	0.001^a^

a Only 1 group of brain region connectivity pass the corrected (*p*<0.05/28, bonferroni corrected).

### 3.5 Correlations of ALFF and ReHo with clinical scale values

The results of Pearson correlation analysis indicated that there was no significant correlation between the ALFF and ReHo values in the aforementioned abnormal brain areas and the severity of insomnia, PSQI, ISI, and HAMD-24 in PI group (*p* > 0.05).

### 3.6 Correlations of PSQI and ISI with HAMD-24

Pearson correlation analysis indicated that the PSQI and ISI scores in the PI group were significantly and positively correlated with the HAMD-24 scores (*p* < 0.05).

## 4 Discussion

Several brain areas within the frontal, parietal, and temporal lobes, together with the cerebellar hemispheres, exhibited significant alterations in ALFF, ReHo, and FC between female PI patients and HC group in the present study. The increase in ALFF and ReHo values may suggest heightened arousal and increased neural activity within this specific brain region, while lower measurement values are indicative of reduced overall activity in the same region. These results validate the anomalies in neuronal activity, coherence, and FC observed in many brain areas in the insomnia population. Insomnia in females may be associated with abnormalities in these brain areas.

Previous studies have specified that the frontal cortex plays a role in higher-order cognitive functions, alertness, and attention (Jiang et al., 2016). It has also been suggested that the frontal cortex regulates both positive and negative emotions in people (Jiang et al., 2016). Altena’s study of frontal cortical regions in insomnia patients confirmed that frontal cortex activity was underactive in people with chronic insomnia and that there was a significant improvement in frontal cortex activity after treatment, suggesting that frontal cortex activity is important during sleep (Altena et al., 2008). Another study that used rs-fMRI to track the ALFF values of healthy individuals after sleep restriction therapy discovered that the bilateral orbitofrontal cortex and dorsolateral prefrontal cortex ALFF values were lower than those of healthy individuals (Gao et al., 2015). Furthermore, Joo et al. discovered that patients with insomnia had considerably higher gray matter concentrations in the left and right dorsolateral prefrontal cortex (left middle frontal gyrus, right superior frontal gyrus, and bilateral inferior frontal gyrus) compared to control subjects (Joo et al., 2013). The researchers also noted a substantial decrease in the volume of gray matter in the medial prefrontal region. The accumulation of negative emotions in patients or the metabolic disruptions caused by chronic insomnia may underlie the decreased activity observed in the prefrontal cortex. Prefrontal cortex hypoactivity probably is a common feature among individuals with insomnia, as supported by this study’s findings that female patients exhibited reduced ALFF and ReHo values in the bilateral superior frontal gyrus. Further supporting the prevalence of reduced prefrontal cortical activity in insomnia patients, a study using [^18^F]-fluorodeoxyglucose positron emission tomography found that patients with insomnia had reduced relative prefrontal cortical metabolism during wakefulness (Nofzinger et al., 2004). The investigators conjectured that the patients’ state of daytime fatigue may be caused by reduced prefrontal cortical activity as a result of sleep inefficiencies. This is in line with the results of the current investigation, which showed that female patients had bilaterally decreased ALFF and ReHo values in the superior frontal gyrus.

Regarding the parietal cortex, a lot of research has been done on the inferior parietal lobule range. The primary node of the default mode network, which is distinguished by increased organismal metabolism and neuronal activity during rest, is the inferior parietal lobule (Raichle, 2015). The restoration of brain energy is one of sleep’s purposes (Krueger et al., 2016). The inferior parietal lobule is classified as having supramarginal and angular gyri, and the right supramarginal gyrus in this investigation both demonstrated considerably lower ALFF values. Using task-state fMRI, Drummond et al. (2013) discovered that patients with insomnia had less activity in task-related working memory areas, such as the bilateral inferior parietal lobule. Li et al. also reported lower spontaneous regional brain activity in the subparietal lobule in patients with insomnia and a negative correlation with PSQI scores (Jiang et al., 2016). Reduced ALFF values in this region imply a lower subparietal lobular neuronal activity, according to Gao et al.’s rs-fMRI monitoring of a population subjected to sleep deprivation (Gao et al., 2015). Moreover, we discovered a significant reduction in ReHo values in the precuneus and ALFF values in the postcentral gyrus, pointing to a broad correlation between parietal activity and the mechanisms underlying insomnia. The precuneus is associated with inhibitory control and visual processing (Wang et al., 2016). In the present study, all subjects had their eyes closed during the MRI examination. Excluding visual effects, we hypothesize that this may be related to reduced inhibitory control in patients with chronic insomnia. Recent studies have shown (Kropf et al., 2019) that the postcentral gyrus, in addition to the perception of somatic sensations, has an important value for emotional processing, such as recognizing the emotional value of a given stimulus, the generation of emotions, and the control of emotions. The postcentral gyrus (Xia et al., 2019) is also a notable brain region for abnormalities in depressed patients. In the present study, we hypothesized that chronic fatigue and low mood were the main reasons for the decline in neuronal activity, and Chou et al. (2021) found that patients with insomnia showed a significant reduction in regional gray matter volume in the postcentral gyrus relative to the normal group, which was correlated with the quality of their sleep, thus indirectly confirming the validity of this hypothesis. In addition, we found lower activity in the precuneus. Therefore, combining the results of previous studies, we suggest that the lower ReHo or ALFF values in the right inferior parietal lobule, precuneus, and postcentral gyrus of patients with chronic insomnia may be due to the disturbed recovery process of the organism caused by poor sleep quality, the accumulation of negative emotions, the decline of somatosensory sensations due to overstimulation, and the impaired structure and function of the parietal lobe.

We similarly found abnormalities in the bilateral anterior cingulate and paracingulate gyrus and the insula. Past studies have suggested that changes in ReHo values in the insula and cingulate gyrus are indicative of abnormal functional changes in emotional circuits. The cingulate gyrus is an important region of the limbic system. It has been shown that the cingulate gyrus is also involved in negative emotions and plays a key role in cognitive functioning in humans (Phan et al., 2002; Pourtois et al., 2010; Shackman et al., 2011). The fMRI studies of anxiety, depression, and schizophrenia have also found abnormal activity in the cingulate gyrus (Davidson et al., 2003; Krausz et al., 2007; Tu et al., 2012). And, most studies have shown that depression is associated with significantly reduced cingulate gyrus activity in depressed patients. Meanwhile, many researchers have confirmed that there are also abnormal changes in the cingulate gyrus and insula in patients with insomnia (Wang et al., 2016). Changes in the lenticular nucleus and putamen have rarely been mentioned before, and our present study proposes that the lentiform nucleus shows low ReHo values (Wang et al., 2016; Zhang et al., 2021). We hypothesize that, as one of the centers in the nervous system that is sensitive to movement and sensation, the poor mood in female insomnia patients is a major factor contributing to the changes in the activity of the lenticular nucleus, putamen, but whether it is a specific brain region for female insomnia needs to be further explored.

We discovered that individuals with insomnia had decreased ALFF values in the angular gyrus, which is consistent with the earlier study by Li et al. on ALFF (Jiang et al., 2016). Additionally, there were elevated ReHo values in the bilateral superior temporal gyrus. These findings suggest a reduction in the activity of the angular gyrus and hyperactivity in the bilateral superior temporal gyrus within the PI group. Dai et al. observed that individuals with insomnia had temporal lobe hyperarousal and proposed that hyperaroused brain areas could also serve as a primary predisposing or persisting factor in chronic insomnia (Dai et al., 2016). Remarkably, these brain areas are associated with emotion as well (Feng et al., 2022).

Changes in the cerebellar hemispheres have recently been incorporated into studies by a growing number of researchers. Negative emotions may also be connected to the cerebellar hemispheres, on the basis of earlier research (Parvizi et al., 2001; Paulus et al., 2004). According to Jung et al., adolescent males’ cerebellar gray matter volume was correlated with more severe insomnia (Jung et al., 2019). In contrast to previous findings, our study revealed an elevation rather than a reduction in the cerebellar hemisphere ALFF values among patients with PI (Dai et al., 2016). We hypothesize that this could be due to different ages of onset or hyperactivation of the region, as well as gender differences. More research is required to determine the precise alterations because the cerebellum has been the subject of few studies and inconsistent perspectives.

Although the presence of depressed patients was excluded from the present study, the HAMD scores of PI group were significantly higher than those of HC group, suggesting that there was some abnormality in the mood of the insomnia patients. Most of the brain regions that showed changes this time were also related to mood, and based on previous reports and our observations of functional changes in mood circuits, we hypothesized that abnormal activity in mood circuits may play an important role in the underlying mechanisms of PI. Moreover, compared with men, female patients had greater mood swings and were more significantly affected by mood. We further hypothesized that female patients with PI have a relatively elevated likelihood of developing depression and anxiety disorders due to changes in brain regions. In the present study, we also found brain region connectivity changes mainly in the superior frontal gyrus, and parietal lobe, and mainly in the decline of FC, which is highly similar to the previous study by Yang et al. (2023). and confirms the rationality and reliability of the present study. The rs-fMRI is widely regarded as an effective modality for investigating neural activity in individuals with insomnia. Previous research on gender differences in PI has predominantly utilized EEG as a primary methodology (Buysse et al., 2008). Even so, the existing literature on gender differences in this area remains limited. We posit that our current findings add important information to the field in several respects.

There are some limitations in this study; the small sample size and the failure to add a male control group or a depressed insomnia group can have an impact on the accuracy of the results. Scale evaluation is limited by the absence of an objective measure for PI; therefore, polysomnography should be utilized instead. We plan to do a follow-up study with a larger sample size to compare patients with and without depression and sleeplessness across genders. Moreover, a more objective assessment method will be employing to appraise the sleep quality of patients.

## 5 Conclusion

In conclusion, our results revealed that the alterations in brain regions of female patients with PI involved multiple functional networks, including the default mode network (SFGmed, IPL.R, TPOsup.R, PCUN.L, and SMG.R, etc.), the salience network (ACG and INS.R etc.), the central executive network, and the limbic network. Reduced coordination between functional networks may be an important mechanism for insomnia and may lead to reduced cognitive function and decision-making ability.

## Data availability statement

The datasets presented in this study can be found in online repositories. The names of the repository/repositories and accession number(s) can be found in the article/supplementary material.

## Ethics statement

The studies involving humans were approved by Ethics Committee of the Affiliated Hospital of Traditional Chinese Medicine of Southwest Medical University. The studies were conducted in accordance with the local legislation and institutional requirements. The participants provided their written informed consent to participate in this study. Written informed consent was obtained from the individual(s) for the publication of any potentially identifiable images or data included in this article.

## Author contributions

HZ: Conceptualization, Data curation, Formal analysis, Investigation, Methodology, Project administration, Resources, Software, Supervision, Validation, Visualization, Writing – original draft, Writing – review & editing. PJ: Investigation, Software, Supervision, Writing – review & editing. YiL: Investigation, Software, Supervision, Writing – review & editing. LW: Conceptualization, Methodology, Software, Supervision, Validation, Visualization, Writing – review & editing. OW: Funding acquisition, Investigation, Software, Supervision, Visualization, Writing – review & editing. YZ: Funding acquisition, Supervision, Writing – review & editing. JF: Software, Supervision, Writing – review & editing, Data curation. QW: Supervision, Writing – review & editing. JZ: Formal analysis, Funding acquisition, Supervision, Writing – review & editing. YoL: Funding acquisition, Writing – review & editing.
